# Prevalence, predictors and outcomes of thyroid dysfunction in patients with acute myocardial infarction: the ThyrAMI-1 study

**DOI:** 10.1007/s40618-020-01408-0

**Published:** 2020-09-08

**Authors:** A. Jabbar, L. Ingoe, H. Thomas, P. Carey, S. Junejo, C. Addison, J. Vernazza, D. Austin, J. P. Greenwood, A. Zaman, S. Razvi

**Affiliations:** 1 Translational and Clinical Research Institute, Faculty of Medical Sciences, Newcastle University, Newcastle upon Tyne, NE1 3BZ UK; 2 Department of Cardiology, Newcastle Upon Tyne Hospitals NHS Foundation Trust, Newcastle upon Tyne, UK; 3 Department of Endocrinology, Gateshead Health NHS Foundation Trust, Gateshead, UK; 4 Department of Cardiology, Northumbria Healthcare NHS Foundation Trust, Cramlington, UK; 5 Department of Endocrinology and Cardiology, South Tyneside and Sunderland NHS Foundation Trust, Sunderland, UK; 6 Department of Biochemistry, South of Tyne Pathology Centre, Gateshead Health NHS Foundation Trust, Gateshead, UK; 7 Department of Cardiology, South Tees Hospitals NHS Foundation Trust, Middlesbrough, UK; 8 Leeds University and Leeds Teaching Hospitals NHS Trust, Leeds, UK

**Keywords:** Subclinical hypothyroidism, Subclinical hyperthyroidism, Low T3 syndrome, Thyroid dysfunction, Acute myocardial infarction, Prevalence and predictors

## Abstract

**Purpose:**

Thyroid dysfunction in patients with cardiac disease is associated with worse outcomes. This study aimed to evaluate the prevalence and analyse predictors and outcomes of thyroid dysfunction in patients presenting with an acute myocardial infarction (AMI).

**Methods:**

A prospective multicentre observational study of patients recruited from six acute hospitals within the North of England. Consecutive patients without previous thyroid disease presenting with both ST-elevation AMI (STEMI) and non-ST-elevation AMI (NSTEMI) were recruited to the Thyroxine in Acute Myocardial Infarction 1 (ThyrAMI-1) cohort study between December 2014 and 2016. Thyroid profile, standard biochemistry measurements and demographic information were obtained within 12 h of admission to hospital. Multivariable logistic regression analyses were performed to assess the predictors of thyroid dysfunction and Cox proportional hazards analyses were utilised to compare all-cause mortality by categories of thyroid dysfunction up to June 2019.

**Results:**

Of the 1802 participants analysed, 1440 (79.9%) were euthyroid, 312 (17.3%) had subclinical hypothyroidism (SCH), 22 (1.2%) had subclinical hyperthyroidism (SHyper) and 25 (1.3%) had low T3 syndrome (LT3S). Predictors for SCH were increasing age, female sex, higher thyroid peroxidase antibody (TPOAb) levels, higher serum creatinine levels and early morning sampling time (between 00:01–06:00 h). The predictors of SHyper were lower body mass index and afternoon sampling time (between 12:01 and 18:00 h). Predictors of LT3S were increasing age, higher creatinine levels and presence of previous ischaemic heart disease. Compared to the euthyroid group, patients with LT3S had higher all-cause mortality; adjusted hazard ratio (95% CI) of 2.02 (1.03–3.95), *p* = 0.04, whereas those with SCH and SHyper did not exhibit significantly increased mortality; adjusted hazard ratios (95% CI) of 1.05 (0.74–1.49), *p* = 0.79 and 0.27 (0.04–1.95), *p* = 0.19, respectively.

**Conclusions:**

Thyroid dysfunction is common in AMI patients on admission to hospital and our data provide an understanding regarding which factors might influence thyroid dysfunction in these patients. Furthermore, the negative association between LT3S and increased mortality post-AMI has once again been highlighted by this study. More research is required to assess if treatment of thyroid dysfunction improves clinical outcomes.

## Background

Thyroid dysfunction is common and can affect between 10–15% of the adult population [1]. Both subclinical hypothyroidism (SCH) and subclinical hyperthyroidism (SHyper) are associated with higher risk of cardiovascular morbidity and mortality [2, 3]. The prevalence of both SCH and SHyper is influenced by age, sex, iodine status and smoking habits [4]. Furthermore, several observational studies have demonstrated that SCH and low circulating triiodothyronine (T3) syndrome (LT3S) are associated with poorer outcomes in patients with acute cardiac conditions [5–8]. However, the prevalence and predictors of thyroid dysfunction in patients with acute myocardial infarction (AMI) remains unclear.

Cardiovascular diseases including AMI remain a leading cause of mortality and morbidity worldwide [9]. Thyroid function tests are frequently requested both in community-living individuals and in hospitalised patients [10]. This understanding may help to prevent inappropriate diagnoses being made and unnecessary treatments being initiated [11]. A previous analysis of participants presenting with AMI demonstrated that sample timing has a significant impact on the diagnosis and prognosis of subclinical thyroid disease (SCTD) [12]. Therefore, it is important to study the prevalence of thyroid dysfunction in AMI patients and understand factors associated with these biochemical abnormalities. We report here the results of our analyses of the prevalence, predictors and mortality outcomes of thyroid dysfunction in patients presenting with an AMI.

## Methods

### Patients

Consecutive patients from six hospitals located in the North of England with both ST-elevation AMI (STEMI) and non-ST-elevation AMI (NSTEMI) presenting between December 2014 and 2016 were recruited to the Thyroxine in Acute Myocardial Infarction 1 (ThyrAMI-1) cohort study. A detailed protocol has been published previously [13]. In brief, the inclusion criteria included any adult above the age of 18 years who was able to provide informed consent and was diagnosed with an AMI. An AMI was defined as chest pain with dynamic ECG changes or increased troponin enzymes based on standard criteria [14]. Patients were excluded from the study if they were unable to provide informed consent, had advanced malignancy (unlikely to survive for more than 6 months), had an alternative explanation for chest pain, or had normal coronary arteries on angiography, or were on medications which can alter thyroid function such as levothyroxine, anti-thyroid drugs, lithium, or amiodarone.

All patients provided written informed consent and the study was approved by the local Research Ethics Committee (REF 14/NE/0151). Participants with confirmed AMI were recruited to the study and had their thyroid profile analysed on the first available sample on admission or, at the latest, within 12 h. More than 98% of patients had their thyroid function tested on the admission sample and the rest (due to lack of adequate quantity of serum) had thyroid function assessed within 6–12 h of admission. Other routine blood parameters such as total cholesterol, peak troponin T or I at 6–12 h post admission and serum creatinine, were also evaluated on admission. In addition, demographic details and clinical information including age, sex, body mass index (BMI), type of AMI (STEMI or NSTEMI), smoking history (categorised as current-, ex- or never-smoker), heart rate, systolic and diastolic blood pressure, and history of existing medical conditions (obtained from medical records) such as ischaemic heart disease, diabetes mellitus, hypertension, hypercholesterolaemia, cerebrovascular disease or atrial fibrillation (all categorised as yes or no) were also noted. Furthermore, as both serum TSH and T3 are known to have a diurnal variation, the time period of blood sampling was also noted (categorised as 00:01–06:00, 06:01–12:00, 12:0–18:00 and 18:01–00:00 h) [15]. Mortality outcomes up to 30th June 2019 were evaluated for each participant via the National Health Service (NHS) Summary Care Records linked through their unique NHS number that provides up to date life status for all registered patients [16].

### Biochemistry

Serum TSH, free thyroxine (FT4), free triiodothyronine (FT3), high sensitivity (hs) troponin T, total cholesterol, creatinine and hs C-reactive protein (CRP) were analysed at four sites using the Roche immunoassay (Roche ecobas, Roche Diagnostics, Medway, Kent, UK). At two other sites, the same analytes were measured using the Advia Centaur immunoassay (Siemens Healthineers, Surrey, UK). Reference ranges were uniformly applied as follows: TSH (0.4–4.0 mIU/L), FT4 (9.0–25.0 pmol/L), FT3 (3.0–7.0 pmol/L), hs troponin T (0–14 ng/L), hs troponin I (0–45 ng/L), creatinine (70–110 μmol/L), total cholesterol (< 4.5 mmol/L). Anti-thyroid peroxidase antibodies (TPOAb) were measured by the Roche immunoassay and levels below 35 mU/L were classed as negative.

Thyroid status was defined based on the following biochemical finding:

Euthyroidism—TSH, FT4 and FT3 levels within their respective reference ranges.

SCH—TSH > 4.0 mIU/L and FT4 levels within the reference range.

SHyper—TSH < 0.4 mIU/L and with FT4 and FT3 levels within the reference range.

Overt hypothyroidism—TSH > 4.0 mIU/L and low FT4 levels.

Overt hyperthyroidism—TSH < 0.4 mIU/L and high FT4 and/or FT3 levels.

LT3S—TSH and FT4 levels within their respective reference ranges, but FT3 < 3.0 pmol/L.

A small number of participants presented with high TSH, normal FT4 and low FT3 levels (*n* = 6) and they were classed as having SCH as serum TSH is widely accepted as being a robust biomarker of thyroid status.

### Statistical analyses

For baseline characteristics, categorical data are expressed as numbers and percentages and compared using the Chi-squared test, whereas continuous variables are expressed as mean ± standard deviation (SD) and compared using ANOVA. Non-parametric continuous variables (such as serum TSH and troponin) were logarithmically transformed prior to analysis.

Both troponin T and I levels are reflective of the severity of myocardial damage and are used to diagnose AMI, but their absolute values differ. Therefore, we first standardised, centred and then combined the two values to form a single hs-standardised Troponin (st Troponin) variable. This combined single variable was utilised in all analyses.

Predictors of thyroid dysfunction (SCH, SHyper and LT3S) were assessed using multivariable logistic regression analysis. Overt hypo- and hyperthyroidism were not analysed due to few patients being classed in this category. The predictor variables included age, sex, body mass index, smoking status, type of AMI, st Troponin, serum creatinine, CRP levels, TPOAb levels, time-period of sampling, and presence of ischaemic heart disease, hypertension, type 2 diabetes mellitus, hypercholesterolaemia, cerebrovascular disease and atrial fibrillation. Missing data were dealt with using multiple imputation method. Ten imputed datasets were created and pooled results were summarised. A sensitivity analysis was performed for predictors of SCH, SHyper and LT3S by analysing the original non-imputed dataset.

The relationship between thyroid dysfunction and all-cause mortality was evaluated using Cox proportional hazards analysis. Survival times were calculated from the date of the AMI till the date of death or date of being known to be alive on the NHS Summary Care Records system. Analyses were adjusted for relevant variables such as age, sex, body mass index, smoking status, type of AMI, st Troponin, serum creatinine, CRP levels, TPOAb levels, and presence of ischaemic heart disease, hypertension, type 2 diabetes mellitus, hypercholesterolaemia, cerebrovascular disease and atrial fibrillation.

A *p* value of < 0.05 was deemed as being indicative of statistical significance. Analyses were performed using the statistical software package SPSS v24 (Ill, Chic, USA).

## Results

### Prevalence of thyroid dysfunction and baseline characteristics

**Fig. 1:**
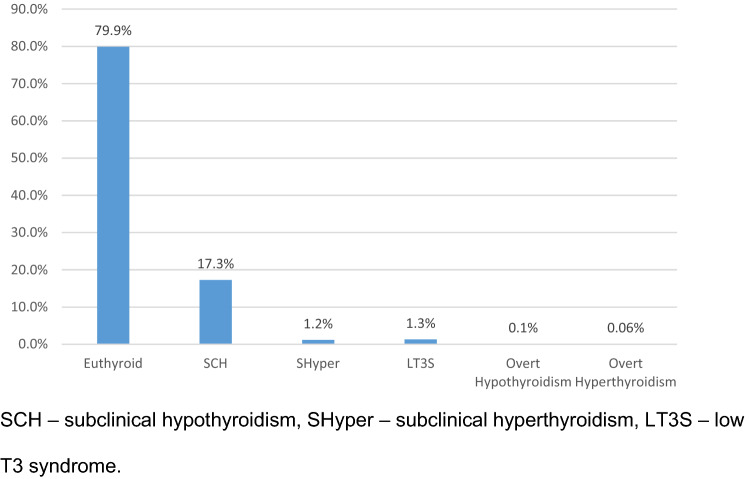
Prevalence of thyroid dysfunction in the ThyrAMI-1 study. *SCH* subclinical hypothyroidism, *SHyper* subclinical hyperthyroidism, *LT3S* low T3 syndrome

Data from 1802 patients were analysed. Of these, 1440 (79.9%) patients were euthyroid, 312 (17.3%) had SCH, 22 (1.2%) had SHyper, 25 (1.3%) had LT3S, 2 (0.1%) had overt hypothyroidism and 1 (0.06%) had overt hyperthyroidism (Fig. 1).

The baseline demographic and clinical characteristics of all participants with euthyroidism, SCH, LT3S and SHyper are outlined in Table 1. The SCH and LT3S patients tended to be older, included a higher percentage of females, had higher serum creatinine levels and standardised troponin levels compared to the euthyroid and SHyper groups. Patients with STEMIs were observed more frequently in the SCH and SHyper groups. Interestingly, patients with LT3S had a higher proportion with existing ischaemic heart disease than the other groups (Table 1). The proportion of SCH patients was highest in the time period 00:01–06:00 h, whereas the proportion of SHyper was highest between 18:01–12:00 h.

**Table 1:** Characteristics of patients presenting with acute myocardial infarction by thyroid status

	Euthyroid (*n* = 1440)	SCH (*n* = 312)	SHyper (*n* = 23)	Low T3 syndrome (*n* = 25)	*p* value
Age (years)	63.4 (± 12.0)	65.8 (± 11.3)	64.3 (± 13.2)	70.9 (± 12.5)	0.001
Males (*n*, %)	1074 (72.7)	213 (68.3)	18 (78.3)	16 (64.0)	0.08
Body mass index (kg/m^2^)	28.5 (± 5.4)	28.4 (± 6.1)	26.2 (± 3.9)	27.9 (± 5.6)	0.25
Systolic BP (mmHg)	141.8 (± 27.4)	140 (± 29.3)	140.9 (± 34.7)	142.9 (± 35.6)	0.42
Diastolic BP (mmHg)	80.3 (± 23.4)	82.1 (± 16.9)	79.4 (± 22.5)	82.9 (± 19.7)	0.48
Heart rate (bpm)	77.1 (± 18.7)	80.3 (± 18.3)	82.1 (± 22.9)	79.6 (± 20.4)	0.32
Current smoker (*n*, %)	457 (31.7)	94 (30.1)	8 (34.8)	9 (36.0)	0.87
Ex-smoker (*n*, %)	449 (31.2)	107 (34.3)	5 (21.7)	7 (28.0)	0.87
STEMI (*n*, %)	695 (48.3)	181 (58.0)	13 (56.5)	11(44.0)	0.01
TSH (mIU/L)	1.8 (1.3–2.5)	5.3 (4.5–6.5)	0.3 (0.3–0.4)	1.6 (1.0–2.9)	< 0.001
FT4 (pmol/L)	16.3 (± 2.9)	15.9 (± 2.9)	16.5 (± 4.5)	13.9 (2.5)	0.007
FT3 (pmol/L)	4.7 (± 0.8)	5.0 (± 1.0)	4.7 (± 0.6)	2.7 (± 0.5)	< 0.001
Positive TPOAb status (> 35 U/L) (*n*, %)	422 (29.3)	164 (52.6)	12 (50.0)	5 (21)	0.05
TPOAb (U/L)	39 (28–190)	128 (28–286)	35 (28–89)	30 (28–120)	< 0.001
hs C-reactive protein (mg/L)	2 (1–9)	1 (1–7)	6 (1–46)	4 (1–52)	0.39
Total Cholesterol (mmol/L)	4.97 (± 1.4)	4.91 (± 1.3)	4.90 (± 1.1)	4.98 (± 1.5)	0.42
Creatinine (µmol/dL)	88.9 (± 33.5)	95.2 (± 66.2)	91.8 (± 29.6)	109.0 (± 59.3)	0.009
Standardised troponin	0.004 (− 0.78–0.88)	0.21 (− 0.59–0.90)	− 0.07 (− 0.83–0.90)	0.22 (− 0.80–0.67)	0.04
Ischaemic heart disease (*n*, %)	373 (25.9)	74 (23.7)	3 (13)	13 (52.0)	0.009
Type 2 diabetes mellitus (*n*, %)	237 (16.5)	55 (17.6)	5 (21.7)	4 (16.0)	0.87
Hypertension (*n*, %)	580 (40.3)	122 (39.1)	9 (39.1)	13 (52.0)	0.66
Hypercholesterolaemia (*n*, %)	369 (25.6)	71 (22.7)	4 (17.4)	5 (20.0)	0.55
Cerebrovascular disease (*n*, %)	65 (4.5)	18 (5.8)	2 (8.7)	2 (8.0)	0.53
Atrial fibrillation (*n*, %)	57 (4)	10 (3.2)	1(4.3)	1(4.0)	0.83
Time period (*n*, %)					
00:01–06:00	236 (16.4)	92 (29.5)	1 (4.3)	5 (20)	< 0.001
06:01–12:00	439 (30.5)	79 (25.3)	4 (17.4)	7 (28)	
12:01–18:00	459 (31.9)	65 (10.8)	14 (60.9)	6 (24)	
18:01–00:00	306 (21.2)	76 (24.4)	4 (17.4)	7 (28)	

*SCH* subclinical hypothyroidism, *SHyper* subclinical hyperthyroidism, *STEMI* ST-elevation myocardial infarction, *TSH* thyrotropin, *FT4* free thyroxine, *FT3* free triiodothyronine, *TPOAb* thyroid peroxidase antibody

Data are presented as mean (± SD), numbers (%) or median (IQR)

### Predictors of thyroid dysfunction

Predictors for SCH were increasing age, female sex, higher TPOAb levels, higher serum creatinine levels and the time of blood sampling (Table 2). With regard to sampling time, patients who had their thyroid function tested between 00:01 and 06:00 h were more likely to have SCH than those sampled at other time points (*p* for trend < 0.001).

**Table 2:** Predictors of SCH in patients with acute myocardial infarction

	Odds ratio (95% CI)	*p* value
Age (years)	1.03 (1.01–1.05)	< 0.001
Sex		
Male	1.00 (Reference)	
Female	1.40 (1.04–1.90)	0.03
Body mass index (kg/m^2^)	1.00 (0.98–1.03)	0.54
Smoking		0.41
Never smoked	1.00 (Reference)	
Current smokers	1.20 (0.83–1.73)	
Ex-smokers	1.22 (0.90–1.69)	
Type of acute myocardial infarction		
NSTEMI	1.00 (Reference)	
STEMI	1.37 (0.98–1.92)	0.06
Standardised Troponin	1.16 (0.99–1.36)	0.07
Creatinine (µmol/dL)	1.00 (1.00–1.01)	0.04
hs CRP (mg/L)	0.99 (0.99–1.00)	0.40
TPOAb (mU/L)	1.01 (1.01–1.03)	< 0.001
Time of sampling (24-h clock)		< 0.001
00:01–06:00	1.00 (Reference)	
06:01–12:00	0.42 (0.29–0.61)	
12.01–18:00	0.32 (0.22–0.47)	
18:01–00:00	0.69 (0.48–0.99)	
Ischaemic heart disease		
Absent	1.00 (Reference)	
Present	1.16 (0.83–1.62)	0.39
Hypertension		
Absent	1.00 (Reference)	
Present	1.14 (0.85–1.53)	0.75
Type 2 diabetes mellitus		
Absent	1.00 (Reference)	
Present	1.08 (0.75–1.57)	0.62
Hypercholesterolaemia		
Absent	1.00 (Reference)	
Present	0.95 (0.68–1.33)	0.64
Cerebrovascular disease		
Absent	1.00 (Reference)	
Present	1.38 (0.77–2.46)	0.21
Atrial fibrillation		
Absent	1.00 (Reference)	
Present	0.63 (0.31–1.30)	0.21

*NSTEMI* Non-ST elevation myocardial infarction, *STEMI* ST elevation myocardial infarction, *hs* highly sensitive, *CRP* C-reactive protein, *TPOAb* Thyroid peroxidase antibody

Significant predictors for SHyper were lower BMI and time of blood sampling (Table 3). With regard to the latter, samples obtained between 00:01 and 06:00 had the least likelihood of being diagnosed with SHyper (*p* for trend 0.02).

**Table 3:** Predictors of Subclinical Hyperthyroidism in patients with acute myocardial infarction

	Odds ratio (95% CI)	*p* value
Age (years)	0.99 (0.95–1.04)	0.68
Gender		
Male	1.0 (Reference)	
Female	1.11 (0.37–3.33)	0.85
Body mass index (kg/m^2^)	0.88 (0.78–0.98)	0.02
Smoking		0.34
Never smoked	1.0 (Reference)	
Current smokers	1.37 (0.34–5.51)	
Ex-smokers	2.29 (0.69–7.60)	
Type of acute myocardial infarction		
NSTEMI	1.0 (Reference)	
STEMI	2.34 (0.80–6.83)	0.12
Standardised Troponin	0.71 (0.43–1.15)	0.17
Creatinine (µmol/dL)	1.00 (0.98–1.02)	0.87
hs CRP (mg/L)	1.00 (0.98–1.02)	0.84
TPOAb (mU/L)	0.99 (0.99–1.00)	0.28
Time of sampling (24-h clock)		0.02
00:01–06:00	1.0 (Reference)	
06:01–12:00	1.60 (0.16–15.7)	
12.01–18:00	6.99 (0.89–55.2)	
18:01–00:00	2.45 (0.25–24.4)	
Ischaemic heart disease		
Absent	1.0 (Reference)	
Present	3.98 (0.84–18.8)	0.07
Hypertension		
Absent	1.0 (Reference)	
Present	1.12 (0.39–3.15)	0.84
Type 2 diabetes mellitus		
Absent	1.0 (Reference)	
Present	2.49 (0.81—7.69)	0.13
Hypercholesterolaemia		
Absent	1.0 (Reference)	
Present	0.53 (0.15–1.96)	0.32
Cerebrovascular disease		
Absent	1.0 (Reference)	
Present	2.92 (0.58–14.7)	0.24
Atrial fibrillation		
Absent	1.0 (Reference)	
Present	–*	0.99

*NSTEMI* Non-ST elevation myocardial infarction, *STEMI* ST elevation myocardial infarction, *hs* highly sensitive, *CRP* C-reactive protein, *TPOAb* Thyroid peroxidase antibody

*Too few to calculate odds ratio

The only significant predictors for LT3S were increasing age, higher creatinine levels and presence of ischaemic heart disease (Table 4). Neither time of sampling, larger infarcts (as measured by peak st troponin levels) or higher CRP levels were significant predictors.

**Table 4:** Predictors of low T3 syndrome in patients with acute myocardial infarction

	Odds ratio (95% CI)	*p* value
Age (years)	1.06 (1.01–1.11)	0.01
Gender		
Male	1.0 (Reference)	
Female	1.60 (0.65–3.93)	0.31
Body mass index (kg/m^2^)	1.03 (0.95–1.11)	0.54
Smoking		0.41
Never smoked	1.0 (Reference)	
Current smokers	3.06 (0.92–10.2)	
Ex-smokers	0.93 (0.33–2.61)	
Type of acute myocardial infarction		
NSTEMI	1.0 (Reference)	
STEMI	1.43 (0.49–4.1)	0.51
Standardised Troponin	1.06 (0.65–1.74)	0.81
Creatinine (µmol/dL)	1.01 (1.00–1.03)	0.03
hs CRP (mg/L)	1.00 (0.99–1.01)	0.42
TPOAb (mU/L)	0.99 (0.99–1.00)	0.75
Time of sampling (24-h clock)		0.34
00:01–06:00	1.0 (Reference)	
06:01–12:00	0.73 (0.22–2.53)	
12.01–18:00	0.61 (0.17–2.10)	
18:01–00:00	1.36 (0.41–4.54)	
Ischaemic heart disease		
Absent	1.0 (Reference)	
Present	2.84 (1.12–7.18)	0.03
Hypertension		
Absent	1.0 (Reference)	
Present	1.43 (0.57–3.59)	0.40
Type 2 diabetes mellitus		
Absent	1.0 (Reference)	
Present	0.50 (0.15–1.66)	0.23
Hypercholesterolaemia		
Absent	1.0 (Reference)	
Present	0.51 (0.17–1.50)	0.24
Cerebrovascular disease		
Absent	1.0 (Reference)	
Present	1.61 (0.34–7.62)	0.57
Atrial fibrillation		
Absent	1.0 (Reference)	
Present	–*	0.99

*NSTEMI* Non-ST elevation myocardial infarction, *STEMI* ST elevation myocardial infarction, *hs* highly sensitive, *CRP* C-reactive protein, *TPOAb* Thyroid peroxidase antibody

*Too few to calculate odds ratio

Several sensitivity analyses were performed. First, the complete case data were analysed without the imputed values. The overall strength and direction of associations remained similar to the main analysis although the parameters of uncertainty (95 percent confidence intervals) were larger (data not shown). Second, to exclude any impact of differences in immunoassays, only patients whose thyroid function and other biochemical parameters were measured using the Roche assay were analysed. These results too were broadly unchanged to the main analysis (data not shown). This second sensitivity analysis also confirmed that combining the standardised values of troponin T and troponin I did not have a stochastically significant impact on the results.

### Thyroid dysfunction and mortality

The median (IQR) follow-up period was 42 (37–49) months. During the follow-up period, there were 156 (10.9%), 43 (13.8%), 1 (4.5%) and 8 (32%) deaths in the euthyroid, SCH, SHyper and LT3S groups, respectively.

**Fig. 2:**
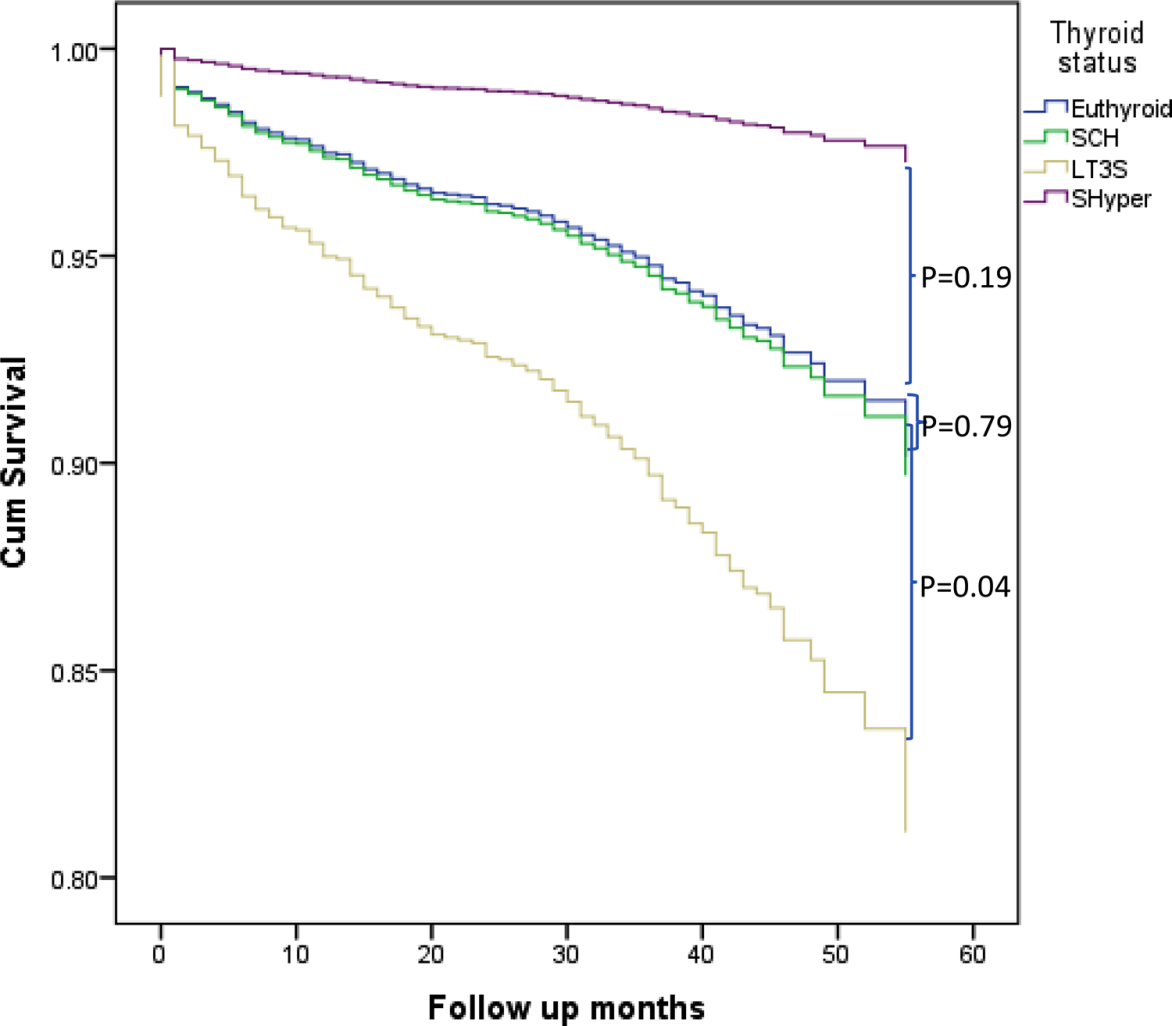
Survival curves to demonstrate the association of thyroid dysfunction with mortality. *SCH* subclinical hypothyroidism, *SHyper* subclinical hyperthyroidism, *LT3S* low T3 syndrome. All-cause mortality in the various thyroid function groups was evaluated using Cox proportional hazards analysis and adjusted for age, sex, body mass index, smoking status, type of AMI, st Troponin, serum creatinine, CRP levels, TPOAb levels, and presence of ischaemic heart disease, hypertension, type 2 diabetes mellitus, hypercholesterolaemia, cerebrovascular disease and atrial fibrillation

Compared to the euthyroid group, participants with SCH and SHyper at baseline were not associated with increased mortality with an adjusted hazard ratio (95% CI) of 1.05 (0.74–1.49), *p* = 0.79 and 0.27 (0.04–1.95), *p* = 0.19, respectively. However, participants with LT3S demonstrated an increased risk of mortality; adjusted hazard ratio (95% CI) of 2.02 (1.03–3.95), *p* = 0.04 (Table 5). Figure 2 demonstrates the survival curve for the thyroid function groups.

**Table 5:** Predictors of long-term mortality in all the patients in the ThyrAMI 1 study using the Cox proportional hazard model

Variable	HR (95% CI)	*p* value
Female gender	0.84 (0.61–1.17)	0.30
Age (per year increase)	1.09 (1.07–1.11)	< 0.001
BMI (per unit increase)	0.99 (0.96–1.02)	0.39
Smoking		
Never smoked	1.0 (Reference)	
Current smokers	1.74 (1.16–2.61)	0.001
Ex-smokers	0.74 (0.53–1.04)	
Type of MI		
NSTEMI	1.49 (1.02–2.19)	0.04
Thyroid status		
Euthyroid	1.0 (Reference)	0.10
SCH	1.05 (0.74–1.49)	
SHyper	0.27 (0.04–1.95)	
LT3S	2.02 (1.03–3.95)	
Standardised troponin (per unit increase)	1.21 (1.01–1.45)	0.042
Creatinine (per µmol/dL increase)	1.004 (1.002–1.006)	< 0.001
CRP (per mg/L increase)	1.008 (1.004–1.013)	< 0.001
TPOAb (per U/L increase)	1.01 (0.99–1.002)	0.35
Ischaemic heart disease	1.16 (0.84–1.60)	0.36
Diabetes mellitus	2.06 (1.490–2.83)	< 0.001
Hypertension	1.004 (0.73–1.37)	0.98
Hypercholesterolaemia	0.71 (0.49–1.02)	0.07
Cerebrovascular disease	1.18 (0.73–1.90)	0.51
Atrial fibrillation	1.88 (1.21–2.91)	0.005

*BMI* body mass index, *MI* myocardial infarction, *NSTEMI* Non-ST elevation myocardial infarction, *TSH* thyrotropin, *FT4* free thyroxine 4, *FT3* free triiodothyronine, *LT4* levothyroxine, *CRP* C-reactive protein

*p < 0.05 indicating statistical significance

The other variables associated with increased mortality included age, current smoking, NSTEMI, standardised troponin, serum creatinine, CRP and presence of diabetes mellitus and atrial fibrillation (Table 5).

## Discussion

This study confirms a high prevalence of thyroid dysfunction in AMI patients. In particular, SCH is the most frequent abnormal thyroid state with almost one in six individuals being diagnosed. The prevalence of LT3S and SHyper is lower with approximately 1 in 100 patients being affected. Importantly, our study also provides information on predictors of these thyroid dysfunction states, which may be useful for clinicians managing patients with AMI.

Several observational studies have shown that the prevalence of thyroid dysfunction in patients with AMI is relatively high [17–19]. The results of this analysis are important for both confirming previously reported prevalence rates and uniquely identifying risk factors for the common thyroid dysfunction states observed in patients with AMI. Older individuals, females, those with higher TPOAb levels or higher creatinine concentrations had a higher prevalence of SCH. Of clinical importance, patients who had their thyroid function samples obtained in the early hours of the morning also had a higher prevalence of SCH. These findings are consistent with previous reports obtained from community-dwelling adults [1, 20, 21]. The NHANES III Study showed females to have higher TSH values as did older individuals and those with positive TPOAb levels [20]. A retrospective database analysis from Tayside, Scotland also reported that the 97.5th TSH centile increases progressively after the age of 40 years [22]. However, this increase in the upper limit of the TSH reference interval with age may not represent true SCH, as it is physiological for serum TSH levels to increase as one gets older [23].

The relationship between TPOAb and SCH is recognised and provides an additional explanation for the increased prevalence of SCH in females. For example, in the NHANES III Study, 60% of SCH cases were associated with elevated TPOAb and such levels were higher in females and increased with age [20]. The Rotterdam Study showed individuals positive for TPOAb to have higher TSH levels and lower T4 concentrations [24]. A 20-year follow-up of the Whickham cohort participants demonstrated that progression of SCH to overt hypothyroidism was dependent not only on the baseline TSH value but also on the presence of thyroid autoimmunity [25].

The relationship between the time of blood sampling and serum TSH variation has been known for some time and the impact on thyroid dysfunction has been previously published from this same patient cohort [12]. In addition, analysis of TSH values in over 400,000 euthyroid individuals showed that a significant nocturnal rise in TSH levels which resulted in the upper limit of the TSH reference interval to increase from 6.45 to 7.55 at midnight [26]. Our data, therefore, confirm SCH to be more prevalent in AMI patients who have their thyroid function tested in early morning than other time periods—due to the physiological circadian rhythm of TSH secretion. Conversely, SHyper is less likely to be diagnosed from samples obtained in the early hours of the morning. These findings are clinically important as they suggest that the time of sampling should be considered when devising the reference range for TSH to avoid inappropriate classification of individuals with thyroid dysfunction.

The present study demonstrates that an increase in creatinine levels is a significant predictor of SCH and low T3, further supporting the view that thyroid dysfunction may be related to renal function. The relationship between elevated TSH levels and kidney disease has previously been investigated with studies showing SCH and overt hypothyroidism to be strongly associated with increased creatinine levels and the progression to chronic kidney disease [27, 28]. Studies have also demonstrated that the decline in kidney function may be reduced by levothyroxine therapy [29, 30]. A possible explanation for the observed relationship between renal impairment and underactive thyroid states include diastolic dysfunction, reduced cardiac output and increased systemic resistance which all result in reduced renal perfusion [31]. Low T3 in kidney disease and AMI may be due to reduced deiodinase activity leading to less conversion of T4 to T3 in chronic disease states [32].

This study demonstrated that AMI patients with low serum T3 levels had a twofold higher risk of mortality, whereas other forms of thyroid dysfunction such as SCH and SHyper had no significant influence on mortality. These results support previous data demonstrating increased long-term mortality in LT3 patients with cardiovascular disease [6, 8, 33–35]. In a previous analysis from the present study, SCH diagnosed with TSH levels higher than the time-period specific reference range, rather than diagnosed using a uniform TSH reference, was associated with increased mortality [12]. The present study differs from that analysis by analysing outcomes for a longer period of time and by evaluating the effects of additional relevant variables on mortality such as CRP and troponin levels.

A recent trial of levothyroxine treatment did not demonstrate any benefit on left ventricular function in SCH patients post AMI [36]. Interestingly, an open-label 6-month trial of oral T3 therapy in 37 patients with STEMI and low serum T3 levels was deemed to be safe although there was no significant improvement in left ventricular function or scar size.[37]. In other clinical trials in patients with LT3S, T3 therapy has shown a modest effect in improving left ventricular function in patients with heart failure [38, 39] and those undergoing surgical revascularisation [40–42].

Our study has several strengths. A relatively large number of patients across multiple sites were systematically studied and various relevant variables that are known to impact thyroid function were analysed. Furthermore, the prospective design of our study meant that all the relevant variables could be collected in a structured manner. In addition, thyroid function tests were obtained on the first available sample on admission and prior to coronary angiography. Thus, it is unlikely that the acute disease itself or the iodine-containing contrast media would have had a significant effect on thyroid function. This probably explains the low prevalence of the LT3S in our patient cohort compared to others [8, 43, 44].

There are some weaknesses too. Two sites used separate immunoassays to measure thyroid function and other biochemical parameters. It is reassuring that TSH levels are similar in these two assays [45]. Moreover, sensitivity analysis conducted on samples all measured by the same assays confirmed the results to be robust. In addition, the number of patients with SHyper and LT3S was low meaning the results obtained in our analysis were less reliable than for SCH.

In conclusion, thyroid dysfunction is relatively common in patients admitted with AMI with SCH being observed in one in six individuals. Other thyroid dysfunction states such as SHyper and LT3S are relatively less frequent. Older individuals and those with higher creatinine levels are both likely to have SCH or LT3S, whereas women, those with higher TPOAb levels and samples obtained in the early hours of the morning are more likely to be diagnosed with SCH. Furthermore, low serum T3 levels were associated with an increased risk of mortality and this represents a potential therapeutic option for such patients presenting with an AMI.

## Data Availability

The data that support the findings of this study are available from Dr Salman Razvi upon reasonable request.
